# Predictors of survival in dedifferentiated liposarcoma: A population-based analysis of the SEER database

**DOI:** 10.1097/MD.0000000000047738

**Published:** 2026-02-28

**Authors:** Akef Obeidat

**Affiliations:** a College of Medicine, Alfaisal University, Riyadh, Saudi Arabia.

**Keywords:** dedifferentiated liposarcoma, predictors, prognosis, SEER, survival

## Abstract

Dedifferentiated liposarcoma (DDLPS) represents a high-grade sarcoma characterized by transition from well-differentiated liposarcoma to non-lipogenic sarcoma. Population-based data examining prognostic factors and treatment outcomes remain limited. This study aimed to identify predictors of survival and evaluate treatment modalities in patients with DDLPS using the Surveillance, Epidemiology, and End Results database. We identified 3962 patients diagnosed with DDLPS between 2000 to 2022 from the Surveillance, Epidemiology, and End Results database. Patient demographics, tumor characteristics, treatment modalities, and survival outcomes were analyzed. Cox proportional hazards regression models were utilized to identify independent prognostic factors for overall survival (OS) and cancer-specific survival. The cohort had a median age of 66 years (interquartile range 57–75), with male predominance (67.5%). Primary tumors were predominantly located in soft tissues (51.4%) and retroperitoneum (36.1%). The median tumor size was 145mm (interquartile range 82–220 mm). Surgery was performed in 85.4% of patients, while radiation therapy and chemotherapy were administered to 33.5% and 18.2%, respectively. Median OS was 54.0 months with 1-year, 2-year, 3-year, and 5-year survival rates of 78.8% (95% confidence intervals [CIs]: 77.5–80.1%), 67.9% (95% CI: 66.4–69.4%), 59.6% (95% CI: 58.0–61.3%), and 47.6% (95% CI: 45.8–49.4%), respectively. In multivariate analysis including 1507 patients with complete staging data, independent predictors of worse OS included advanced age (hazard ratios [HR] = 1.42 per 10 years, 95% CI: 1.34–1.50, *P* < .0001), regional stage (HR = 1.30, 95% CI: 1.13–1.48, *P* < .05), distant stage (HR = 2.45, 95% CI: 2.02–2.97, *P* < .0001), and increasing tumor size (HR = 1.01 per cm, 95% CI: 1.01–1.01, *P* < .0001). Surgical resection conferred significant survival benefit (HR = 0.29, 95% CI: 0.23–0.36, *P* < .0001), as did radiation therapy (HR = 0.75, 95% CI: 0.66–0.86, *P* < .0001). Paradoxically, chemotherapy was associated with worse survival (HR = 1.42, 95% CI: 1.20–1.69, *P* < .0001). Retroperitoneal location demonstrated worse prognosis compared to non-retroperitoneal sites (median OS: 46.0 vs 60.0 months, log-rank *P* = .0001; median cancer-specific survival: 84.0 vs 122.0 months, log-rank *P* < .0001). This large population-based analysis demonstrates that surgical resection remains the cornerstone of treatment for DDLPS, providing substantial survival benefit. Advanced stage, older age, and larger tumor size independently predict poor outcomes. The adverse association with chemotherapy likely reflects selection bias, with systemic therapy reserved for aggressive or advanced disease.

## 1. Introduction

Dedifferentiated liposarcoma (DDLPS) represents a distinct pathological entity characterized by the progression of well-differentiated liposarcoma to non-lipogenic sarcoma, typically exhibiting high-grade morphology reminiscent of undifferentiated pleomorphic sarcoma or myxofibrosarcoma.^[1]^ First described by Evans in 1979,^[2]^ this tumor accounts for approximately 10% of all liposarcomas and carries a significantly worse prognosis compared to its well-differentiated counterpart.^[3]^ The dedifferentiation process can occur de novo in primary tumors or manifest during disease recurrence, with molecular hallmarks including amplification of chromosome 12q13-15 containing *MDM2* and *CDK4* genes.^[4–6]^

The clinical behavior of DDLPS remains heterogeneous and challenging to predict. While local recurrence rates approach 40% to 60%, distant metastasis occurs in 15% to 30% of cases, contrasting sharply with well-differentiated liposarcoma which rarely metastasizes.^[7,8]^ Retroperitoneal location, which accounts for a significant number of cases, presents particular therapeutic challenges due to anatomical constraints limiting complete surgical resection and the propensity for multifocal disease.^[9,10]^ Contemporary management relies primarily on surgical excision, yet the role of adjuvant therapies remains contentious, with conflicting evidence regarding the efficacy of radiation therapy and systemic chemotherapy.^[11,12]^

Population-based analyses examining prognostic factors and treatment outcomes in DDLPS remain scarce, with existing literature predominantly consisting of single-institution retrospective series with inherent selection bias and limited generalizability.^[13]^ The heterogeneity in reporting standards, variability in treatment approaches, and evolution of diagnostic criteria over time further complicate systematic evaluation of this rare malignancy. Previous Surveillance, Epidemiology, and End Results (SEER)-based studies have either grouped DDLPS with other high-grade sarcomas or focused on anatomic site-specific analyses, precluding comprehensive assessment of this specific histological subtype.^[14]^

The present investigation leverages the SEER database to conduct the largest population-based analysis of DDLPS to date. By examining a cohort spanning over 2 decades, this study aims to delineate demographic patterns, characterize treatment utilization, identify independent prognostic factors, and evaluate the impact of multimodal therapy on survival outcomes. These findings may inform risk stratification, guide therapeutic decision-making, and identify areas requiring further investigation in the management of this aggressive sarcoma.

## 2. Methods

### 2.1. Data source and study population

This retrospective cohort study utilized the SEER 18 Registries Database (November 2023 submission), encompassing approximately 28% of the United States population. The SEER program collects comprehensive cancer incidence and survival data from population-based cancer registries, providing high-quality, standardized information with rigorous quality control measures. Given the de-identified nature of publicly available SEER data, institutional review board approval was not required.

### 2.2. Patient selection and eligibility criteria

Patients diagnosed with DDLPS between January 1, 2000, and December 31, 2022, were identified using International Classification of Diseases for Oncology, Third Edition histology code 8858/3. Inclusion criteria comprised: histologically confirmed DDLPS as the primary malignancy or first malignant primary tumor; complete survival data including vital status and follow-up duration; known age at diagnosis. Exclusion criteria included: diagnosis obtained solely from autopsy or death certificate; unknown survival time; patients with zero survival months where death occurred on the date of diagnosis, potentially representing incomplete data capture; other histological forms of liposarcoma, as the study solely focused on the dedifferentiated subtype.

### 2.3. Variable definitions and data processing

Demographic variables extracted included age at diagnosis (analyzed as continuous and categorical: <40, 40–60, 61–80, and >80 years), sex, race/ethnicity (White, Black, Asian/Pacific Islander, American Indian/Alaska Native, and unknown), and year of diagnosis. Tumor characteristics encompassed primary site (categorized using SEER Site Recode International Classification of Diseases for Oncology, Third Edition/WHO 2008), tumor size (continuous in millimeters and categorical: ≤5, 5.1–10, 10.1–20, and >20 cm), histological grade (well, moderately, poorly differentiated, undifferentiated/anaplastic, and unknown), and SEER historic stage (localized, regional, distant, and unstaged).

Treatment modalities were classified as surgery (no surgery vs surgery performed, including codes 10–90 from “RX Summ–Surg Prim Site”), radiation therapy (none vs beam radiation or other radiation modalities), and chemotherapy (no/unknown vs yes). Combined treatment modalities were categorized as surgery alone, surgery plus radiation, surgery plus chemotherapy, trimodality therapy (surgery plus radiation plus chemotherapy), radiation alone, chemotherapy alone, combined radiation and chemotherapy without surgery, or no documented cancer-directed therapy.

### 2.4. Statistical analysis

Descriptive statistics were calculated for all variables, with continuous variables reported as means with standard deviations or medians with interquartile ranges (IQR) based on distribution normality. Categorical variables were expressed as frequencies and percentages. Overall survival was defined as time from diagnosis to death from any cause, while cancer-specific survival (CSS) represented time from diagnosis to death attributed to DDLPS. Patients alive at last follow-up or those who died from other causes (for CSS analysis) were censored. Kaplan–Meier methodology was employed to estimate survival probabilities, with survival rates calculated at 1, 2, 3, and 5 years.

Univariate Cox proportional hazards regression models were constructed to evaluate the association between individual variables and survival outcomes. Variables demonstrating statistical significance at *P* < .1 in univariate analysis were incorporated into multivariate Cox models to identify independent prognostic factors. Given substantial missing data for stage (52.7% missing) and grade (46.8% missing), 2 multivariate models were developed: Model 1 including stage variables with complete case analysis, and Model 2 excluding stage to maximize sample size and statistical power. Hazard ratios (HR) with 95% confidence intervals (CI) were calculated, with continuous variables scaled for clinical interpretability (age per 10 years, year of diagnosis per decade, and tumor size per centimeter).

Model performance was assessed using Harrell concordance index (C-index), with values >0.7 indicating good discrimination. The proportional hazards assumption was verified through examination of Schoenfeld residuals. Subgroup analyses examined survival differences between retroperitoneal and non-retroperitoneal tumors using log-rank testing. Collinearity was assessed using variance inflation factors, with values >10 suggesting multicollinearity requiring variable exclusion or transformation.

To address potential immortal time bias in treatment effect estimates, sensitivity analyses were performed restricting the cohort to patients surviving at least 3 months post-diagnosis. Additional sensitivity analyses examined temporal trends by stratifying outcomes by diagnosis era (2000–2009, 2010–2019, and 2020–2022). All statistical tests were two-sided with significance defined as *P* <.05. Analyses were performed using Python 3.12 with lifelines 0.30.0 for survival analysis, pandas 2.3.3 for data manipulation, and scipy 1.16.2 for statistical testing.

## 3. Results

### 3.1. Patient demographics and clinical characteristics

The final analytic cohort comprised 3962 patients with DDLPS, representing complete survival data from an initial population of 3990 cases (Table 1). The median age at diagnosis was 66 years (IQR 57–75 years, range 12–90 years), with the majority of patients falling within the 61 to 80 year age group (n = 2083, 52.6%). A distinct male predominance was observed, with 2674 males (67.5%) versus 1288 females (32.5%), yielding a male-to-female ratio of 2.1:1. The racial distribution demonstrated White predominance (n = 3269, 82.5%), followed by Asian/Pacific Islander (n = 393, 9.9%), Black (n = 247, 6.2%), and American Indian/Alaska Native populations (n = 33, 0.8%). Temporal analysis revealed increasing diagnosis frequency over the study period, with 912 cases (23.0%) diagnosed in 2000 to 2009, 2104 cases (53.1%) in 2010 to 2019, and 946 cases (23.9%) in 2020 to 2022.

**Table 1:** Patient demographics and clinical characteristics (N = 3962).

Characteristic	n (%) or median (IQR)
Age (yr)	
Median (IQR)	66 (57–75)
<40	156 (3.9)
40–60	1227 (31.0)
61–80	2083 (52.6)
>80	496 (12.5)
Male	2674 (67.5)
Race	
White	3269 (82.5)
Asian/Pacific Islander	393 (9.9)
Black	247 (6.2)
American Indian/Alaska Native	33 (0.8)
Unknown	20 (0.5)
Year of diagnosis	
2000–2009	912 (23.0)
2010–2019	2104 (53.1)
2020–2022	946 (23.9)

IQR = interquartile range.

### 3.2. Tumor characteristics and anatomic distribution

Primary tumor sites demonstrated distinct anatomic predilection, with soft tissues including heart comprising 51.4% (n = 2036) of cases, followed by retroperitoneum at 36.1% (n = 1431) (Table 2). Less common sites included other male genital organs (4.4%), peritoneum/omentum/mesentery (1.8%), kidney and renal pelvis (1.7%), testis (1.4%), and mediastinum (0.7%).

**Table 2:** Tumor characteristics.

Characteristic	n (%) or median (IQR)
Primary site	
Soft tissue including heart	2036 (51.4)
Retroperitoneum	1431 (36.1)
Other male genital organs	175 (4.4)
Peritoneum/omentum/mesentery	72 (1.8)
Kidney and renal pelvis	68 (1.7)
Testis	56 (1.4)
Mediastinum	26 (0.7)
Other sites	98 (2.5)
SEER historic stage*	
Localized	907 (48.4)
Regional	736 (39.3)
Distant	232 (12.4)
Unknown/unstaged	2087
Tumor size (mm)†	
Median (IQR)	145 (82–220)
≤50	407 (12.0)
51–100	728 (21.4)
101–200	1288 (37.9)
>200	974 (28.7)

IQR = interquartile range, SEER = Surveillance, Epidemiology, and End Results.

* Available for 1875 patients (47.3%).

† Available for 3397 patients (85.7%).

Among the 1875 patients (47.3%) with available staging data, localized disease was present in 907 cases (48.4%), regional extension in 736 cases (39.3%), and distant metastasis in 232 cases (12.4%). Tumor size data, available for 3397 patients (85.7%), revealed a median of 145.0 mm (IQR 82–220 mm). Size distribution analysis demonstrated 407 tumors (12.0%) measuring ≤5 cm, 728 (21.4%) measuring 5.1 to 10 cm, 1288 (37.9%) measuring 10.1 to 20 cm, and 974 (28.7%) exceeding 20 cm.

Histological grade information, available for 2108 patients (53.2%), paradoxically indicated well-differentiated histology in all cases with documented grade. This apparent inconsistency likely reflects coding limitations within SEER, as DDLPS by definition contains high-grade components. The substantial missing grade data (46.8%) further suggests challenges in standardized pathological reporting for this entity.

### 3.3. Treatment modalities and therapeutic combinations

Surgical resection constituted the primary treatment modality, performed in 3383 patients (85.4%), while 567 patients (14.3%) received no surgical intervention (Table 3). The high surgical rate reflects the central role of operative management, though the substantial minority without surgery suggests technical unresectability, patient comorbidities, or advanced disease precluding surgical benefit.

**Table 3:** Treatment modalities.

Treatment	n (%)
Surgery	
Performed	3383 (85.4)
Not performed	567 (14.3)
Unknown	12 (0.3)
Radiation therapy	
Given	1329 (33.5)
Not given	2505 (63.2)
Unknown	128 (3.2)
Chemotherapy	
Given	720 (18.2)
Not given/unknown	3242 (81.8)
Treatment combinations	
Surgery alone	1865 (47.1)
Surgery + radiation	1008 (25.4)
Surgery + chemotherapy	324 (8.2)
Surgery + radiation + chemotherapy	186 (4.7)
Chemotherapy alone	165 (4.2)
Radiation alone	90 (2.3)
Radiation + chemotherapy (no surgery)	45 (1.1)
None/unknown	279 (7.0)

Radiation therapy was administered to 1329 patients (33.5%), with 2505 patients (63.2%) receiving no radiation. Chemotherapy utilization was less frequent, delivered to 720 patients (18.2%) versus 3242 (81.8%) without systemic therapy. These patterns align with existing guidelines prioritizing local control through surgery and selective use of adjuvant therapies.

Analysis of treatment combinations revealed surgery alone as the most common approach (n = 1865, 47.1%), followed by surgery plus radiation (n = 1008, 25.4%). Notably, 279 patients (7.0%) received no documented cancer-directed therapy or had unknown treatment status. Trimodality therapy combining surgery, radiation, and chemotherapy was employed in 186 patients (4.7%), while chemotherapy alone (n = 165, 4.2%), radiation alone (n = 90, 2.3%), and combined radiation-chemotherapy without surgery (n = 45, 1.1%) represented less common strategies. These treatment patterns reflect individualized decision-making based on tumor location, resectability, and patient factors.

### 3.4. Survival outcomes and prognostic factors

**Figure 1.**
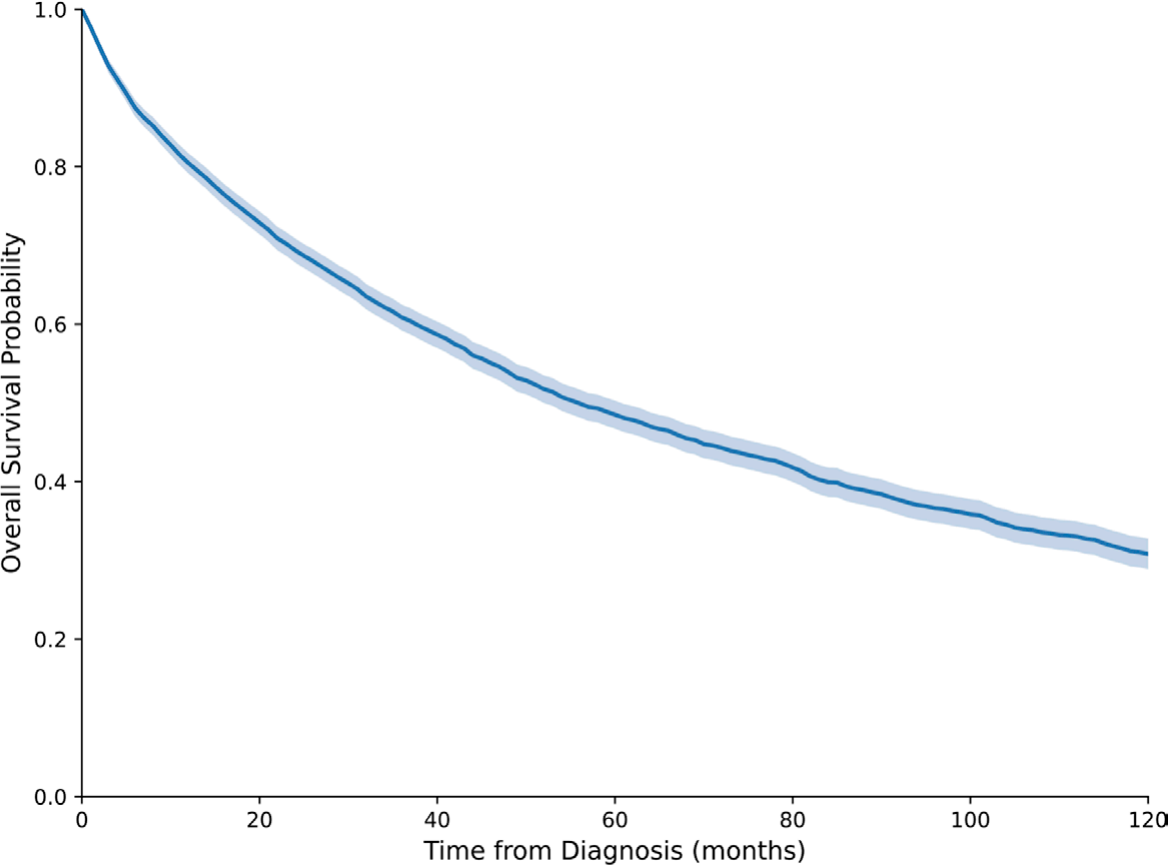
Kaplan–Meir curve demonstrating the overall survival of dedifferentiated liposarcoma patients in the United States.

The median overall survival (OS) was 54.0 months, with Kaplan–Meier estimates revealing 1-year survival of 78.8% (95% CI: 77.5–80.1%), 2-year survival of 67.9% (95% CI: 66.4–69.4%), 3-year survival of 59.6% (95% CI: 58.0–61.3%), and 5-year survival of 47.6% (95% CI: 45.8–49.4%) (Fig. 1). Median CSS was 105.0 months, with 1-year, 3-year, and 5-year CSS rates of 83.4% (95% CI: 82.2–84.6%), 68.8% (95% CI: 67.2–70.4%), and 59.9% (95% CI: 58.1–61.7%), respectively.

Univariate Cox regression identified multiple significant prognostic factors (Table 4). Age demonstrated a consistent adverse effect (HR = 1.37 per 10 years, 95% CI: 1.32–1.41, *P* < .0001), with patients >80 years experiencing particularly poor outcomes (HR = 2.18, 95% CI: 1.91–2.49, *P* < .0001) compared to those <40 years. Year of diagnosis showed worsening outcomes over time in univariate analysis (HR = 1.14 per decade, 95% CI: 1.05–1.23, *P* = .001), though this effect attenuated in multivariate models.

**Table 4:** Univariate Cox regression analysis for overall survival.

Variable	HR (95% CI)	*P*-value
Age (per 10 yr)	1.37 (1.32–1.41)	<.0001
Female (vs male)	0.96 (0.88–1.05)	.390
Year of diagnosis (per decade)	1.14 (1.05–1.23)	.001
Stage (vs localized)		
Regional	1.17 (1.05–1.30)	.005
Distant	2.62 (2.26–3.03)	<.0001
Tumor size (per cm)	1.01 (1.01–1.01)	<.0001
Surgery performed	0.23 (0.20–0.25)	<.0001
Radiation given	0.65 (0.59–0.71)	<.0001
Chemotherapy given	1.59 (1.43–1.76)	<.0001
Retroperitoneal site	1.19 (1.09–1.29)	.0001

Stage powerfully predicted survival, with regional disease conferring HR = 1.17 (95% CI: 1.05–1.30, *P* = .005) and distant metastasis HR = 2.62 (95% CI: 2.26–3.03, *P* < .0001) compared to localized disease. Tumor size demonstrated a linear relationship with mortality risk (HR = 1.01 per cm, 95% CI: 1.01–1.01, *P* < .0001). Surgical resection provided substantial protective effect (HR = 0.23, 95% CI: 0.20–0.25, *P* < .0001), as did radiation therapy (HR = 0.65, 95% CI: 0.59–0.71, *P* < .0001). Conversely, chemotherapy administration associated with increased mortality (HR = 1.59, 95% CI: 1.43–1.76, *P* < .0001) in univariate analysis.

### 3.5. Multivariate analysis and independent predictors

Multivariate Model 1, incorporating 1507 patients with complete staging data (1079 events), identified several independent prognostic factors (Table 5). Advanced age remained significantly associated with mortality (HR = 1.42 per 10 years, 95% CI: 1.34–1.50, *P* < .0001), as did regional stage (HR = 1.30, 95% CI: 1.13–1.48, *P* < .05) and distant stage (HR = 2.45, 95% CI: 2.02–2.97, *P* < .0001). Increasing tumor size maintained independent prognostic significance (HR = 1.01 per cm, 95% CI: 1.01–1.01, *P* < .0001).

**Table 5:** Multivariate Cox regression analysis for overall survival.

Variable	Model 1* (with stage) HR (95% CI)	Model 2† (without stage) HR (95% CI)
Age (per 10 yr)	1.42 (1.34–1.50)‡	1.34 (1.29–1.40)‡
Female (vs male)	0.93 (0.82–1.06)	0.92 (0.84–1.02)
Year (per decade from 2000)	1.07 (0.89–1.29)	0.93 (0.84–1.04)
Regional stage (vs localized)	1.30 (1.13–1.48)‡	–
Distant stage (vs localized)	2.45 (2.02–2.97)‡	–
Tumor size (per cm)	1.01 (1.01–1.01)‡	1.01 (1.01–1.02)‡
Surgery performed	0.29 (0.23–0.36)‡	0.21 (0.18–0.24)‡
Radiation given	0.75 (0.66–0.86)‡	0.69 (0.63–0.77)‡
Chemotherapy given	1.42 (1.20–1.69)‡	1.31 (1.16–1.49)‡
Retroperitoneal site	1.12 (0.99–1.28)	1.12 (1.02–1.24)‡
Model performance		
C-index	0.695	0.715
N (events)	1507 (1079)	3397 (1814)

* Model 1 includes patients with complete staging data.

† Model 2 excludes stage to maximize sample size.

‡ *P* < .05.

Surgical resection emerged as the strongest favorable prognostic factor (HR = 0.29, 95% CI: 0.23–0.36, *P* < .0001), representing a 71% reduction in mortality risk. Radiation therapy conferred additional survival benefit (HR = 0.75, 95% CI: 0.66–0.86, *P* < .0001). Paradoxically, chemotherapy remained associated with worse outcomes after adjustment (HR = 1.42, 95% CI: 1.20–1.69, *P* < .0001). The model demonstrated good discrimination with C-index of 0.695.

Model 2, excluding stage variables to maximize sample size (n = 3397, 1814 events), yielded consistent findings (Table 5). Age (HR = 1.34, 95% CI: 1.29–1.40, *P* < .0001), tumor size (HR = 1.01, 95% CI: 1.01–1.02, *P* < .0001), surgery (HR = 0.21, 95% CI: 0.18–0.24, *P* < .0001), radiation (HR = 0.69, 95% CI: 0.63–0.77, *P* < .0001), and chemotherapy (HR = 1.31, 95% CI: 1.16–1.49, *P* < .0001) maintained similar effect estimates. Notably, retroperitoneal location achieved independent significance in Model 2 (HR = 1.12, 95% CI: 1.02–1.24, *P* < .05). The C-index of 0.715 indicated good model performance without staging information.

### 3.6. Anatomic site-specific outcomes

Subgroup analysis comparing retroperitoneal (n = 1431) versus non-retroperitoneal (n = 2531) tumors revealed significant prognostic differences (Table S1, Supplemental Digital Content, https://links.lww.com/MD/R396). Retroperitoneal tumors demonstrated inferior median OS of 46 months compared to 60 months for non-retroperitoneal sites (log-rank *P* = .0001), and inferior median CSS of 84 months versus 122 months (log-rank *P* < .0001) (Table 6). The retroperitoneal cohort experienced 837 deaths (58.5%) versus 1388 deaths (54.8%) in non-retroperitoneal locations.

**Table 6:** Survival outcomes.

Outcome	Value (95% CI)
Overall survival	
Median (mo)	54.0
1-year survival	78.8% (77.5–80.1)
2-year survival	67.9% (66.4–69.4)
3-year survival	59.6% (58.0–61.3)
5-year survival	47.6% (45.8–49.4)
Cancer-specific survival	
Median (mo)	105.0
1-year survival	83.4% (82.2–84.6)
2-year survival	75.5% (74.0–76.8)
3-year survival	68.8% (67.2–70.4)
5-year survival	59.9% (58.1–61.7)
Site-specific median overall survival	
Retroperitoneal	46.0 months
Non-retroperitoneal	60.0 months
Log-rank *P*-value	.0001
Site-specific median cancer-specific survival	
Retroperitoneal	84.0 months
Non-retroperitoneal	122.0 months
Log-rank *P*-value	<.0001

### 3.7. Temporal trends and era-specific outcomes

Analysis by diagnosis era revealed evolving treatment patterns and outcomes. Surgical rates remained stable (82.1% in 2000–2009, 83.3% in 2010–2019, 83.5% in 2020–2022, *P* = .62), while radiation therapy utilization increased modestly (30.8%, 34.5%, and 35.1%, *P* = .04). Chemotherapy use demonstrated the most substantial change, increasing from 14.3% in 2000 to 2009, 19.5% in 2010 to 2019, and 19.9% in 2020 to 2022 (*P* = .001).

Despite therapeutic advances, crude survival improvements were modest. Median OS increased from 27 months in 2000 to 2009 to 31 months in 2010 to 2019, with insufficient follow-up for the 2020 to 2022 cohort. Year of diagnosis did not achieve statistical significance in multivariate analysis (HR = 1.07, 95% CI: 0.89–1.29).

## 4. Discussion

This population-based analysis of 3962 patients with DDLPS represents the largest cohort study examining this aggressive sarcoma subtype to date, providing critical insights into demographic patterns, treatment utilization, and prognostic determinants. The observed median OS of 54 months and 5-year survival rate of 47.6% underscore the aggressive nature of DDLPS, contrasting sharply with the indolent course of well-differentiated liposarcoma where 5-year survival exceeds 90% for localized disease.^[15]^ These survival statistics are consistent with or slightly exceed institutional series reporting median survival ranging from 24 to 48 months, though direct comparisons are complicated by heterogeneous patient populations and variable inclusion criteria.^[16,17]^

The demographic profile revealing male predominance (2.1:1 ratio) and median age of 66 years corroborates previous reports suggesting hormonal or genetic factors influencing DDLPS development.^[18]^ The age distribution, with 65.1% of patients exceeding 60 years, presents particular therapeutic challenges given competing comorbidities and reduced tolerance for aggressive multimodal therapy. Notably, the independent adverse prognostic impact of age persisted after adjustment for stage and treatment, suggesting age-related biological factors beyond simple treatment selection bias.

Anatomic distribution analysis confirmed retroperitoneal predominance (36.1%) characteristic of DDLPS, though the proportion was lower than some surgical series reporting 50% to 70% retroperitoneal location.^[7]^ This discrepancy likely reflects referral bias in specialized sarcoma centers receiving complex retroperitoneal cases. The inferior outcomes for retroperitoneal tumors (median OS 46.0 vs 60.0 months) primarily reflected larger tumor size and advanced stage at presentation rather than intrinsic biological differences. Retroperitoneal tumors presented with mean size of 205.0 versus 144.5 mm for other sites (*P* < .0001). Notably, retroperitoneal tumors demonstrated higher surgical resection rates (87.8% vs 84.0%, *P* = .002), suggesting that despite more aggressive surgical management, the larger tumor burden and advanced disease at presentation continue to drive inferior outcomes. Retroperitoneal location achieved marginal significance in Model 1 (HR = 1.12, 95% CI: 0.99–1.28) and independent significance in Model 2 (HR = 1.12, 95% CI: 1.02–1.24, *P* < .05). These findings support aggressive surgical approaches in retroperitoneal disease, consistent with recent advocacy for compartmental resection in retroperitoneal sarcoma.^[19–21]^

Surgical resection emerged as the most powerful therapeutic intervention, conferring mortality reduction in multivariate analysis. This dramatic benefit exceeds that reported for most solid malignancies and emphasizes surgery’s central role despite the challenges posed by large tumor size and anatomically complex locations. The surgical rate in this population-based cohort approaches that of specialized centers, suggesting appropriate recognition of surgery’s importance across practice settings. However, the patients receiving no surgical intervention highlights persistent challenges with technical unresectability, medical inoperability, or nihilistic approaches to advanced disease.

The protective effect of radiation therapy supports its selective use, particularly given retroperitoneal predominance where local recurrence represents the primary failure pattern. This finding contrasts with the STRASS trial showing no survival benefit for preoperative radiation in retroperitoneal sarcoma, though DDLPS comprised only a subset of that study population.^[22]^ The radiation utilization rate suggests judicious selection, likely favoring patients with high-risk features including close margins, recurrent disease, or unresectable tumors. The survival benefit observed here may reflect selection of radiation-responsive cases or improved local control translating to survival advantages not captured in randomized trials with strict eligibility criteria.

The paradoxical association between chemotherapy and increased mortality merits careful interpretation. This finding almost certainly represents confounding by indication, with systemic therapy reserved for aggressive, advanced, or recurrent disease carrying inherent poor prognosis. The inability to capture disease burden, performance status, and chemotherapy intent (neoadjuvant, adjuvant, palliative) within SEER limits definitive conclusions about chemotherapy efficacy. Nevertheless, the persistent adverse association after adjusting for stage and other factors suggests limited effectiveness of conventional chemotherapy in DDLPS, consistent with response rates below 20% reported in clinical trials.^[23,24]^

Year of diagnosis did not achieve independent significance in multivariate analysis, suggesting that improvements in outcomes may be mediated through better patient selection and stage-related factors rather than direct therapeutic advances. This temporal trend parallels improvements observed across sarcoma subtypes and may reflect centralization of care in specialized centers, enhanced imaging enabling better staging, and molecular diagnostics improving diagnostic accuracy.^[25]^

Several limitations warrant consideration when interpreting these findings. The retrospective design introduces inherent selection bias and unmeasured confounding. SEER lacks granular clinical data including performance status, comorbidities, margin status, recurrence patterns, and specific chemotherapy regimens, precluding comprehensive risk adjustment. The substantial missing data for stage and grade necessitated multiple analytical approaches and may introduce bias if missingness correlates with unmeasured prognostic factors. Coding inconsistencies, exemplified by all graded tumors classified as well-differentiated despite dedifferentiated histology, suggest data quality issues requiring cautious interpretation. Additionally, the lack of distinction between a patient not receiving chemotherapy and radiotherapy versus an unknown status can add bias to the analysis, as well as lack of granular details on the type of treatment received. The inability to distinguish planned multimodal therapy from salvage treatment further complicates therapeutic effectiveness assessment.

Despite these limitations, this study’s strengths include the large sample size providing statistical power for multivariable modeling, population-based design enhancing generalizability, long follow-up enabling meaningful survival analysis, and contemporary cohort reflecting current diagnostic and therapeutic standards. The concordance indices exceeding 0.7 indicate good model discrimination, supporting the identified prognostic factors’ validity.

Clinical implications of these findings include reinforcement of surgical resection as the therapeutic cornerstone, supporting aggressive attempts at complete excision when technically feasible. The survival benefit associated with radiation therapy suggests its consideration for high-risk cases, though optimal timing and technique require further investigation. The apparent lack of chemotherapy benefit using conventional regimens highlights the urgent need for novel systemic approaches, potentially including targeted therapy against MDM2/CDK4 or immunotherapy strategies.^[26,27]^

Future research directions should prioritize prospective registries capturing detailed clinical parameters, molecular profiling to identify targetable alterations and predict treatment response, clinical trials evaluating novel agents specifically in DDLPS, and comparative effectiveness research examining neoadjuvant versus adjuvant strategies. International collaboration will prove essential given disease rarity, with standardized reporting enabling meaningful cross-institutional comparisons.

## 5. Conclusions

This comprehensive population-based analysis of DDLPS demonstrates the aggressive nature of this sarcoma subtype, with median OS of 54 months despite multimodal therapy. Surgical resection provides paramount survival benefit, reducing mortality risk significantly, while radiation therapy confers additional advantage in selected patients. Advanced age, stage, and tumor size independently predict adverse outcomes, enabling risk stratification. The limited apparent effectiveness of conventional chemotherapy underscores the critical need for novel therapeutic approaches. These findings provide benchmarks for clinical trial design, support aggressive surgical management when feasible, and highlight DDLPS as a high-priority target for innovative treatment strategies. Continued research leveraging molecular insights and immunotherapeutic advances offers hope for improving outcomes in this challenging malignancy.

## Author contributions

**Conceptualization:** Akef Obeidat.

**Formal analysis:** Akef Obeidat.

**Project administration:** Akef Obeidat.

**Writing – original draft:** Akef Obeidat.

**Writing – review & editing:** Akef Obeidat.

## Supplementary Material



## References

[1] Nascimento AG. Dedifferentiated liposarcoma. Semin Diagn Pathol. 2001;18:263–6. 11757866

[2] Evans HL. Liposarcoma A study of 55 cases with a reassessment of its classification. Am J Surg Pathol. 1979;3:507–23. 534388 10.1097/00000478-197912000-00004

[3] Schaefer I-M Fletcher CDM. Diagnostically challenging spindle cell neoplasms of the retroperitoneum. Surg Pathol Clinics. 2015;8:353–74. 10.1016/j.path.2015.05.007 26297061

[4] Binh MB Sastre-Garau X Guillou L. MDM2 and CDK4 immunostainings are useful adjuncts in diagnosing well-differentiated and dedifferentiated liposarcoma subtypes: a comparative analysis of 559 soft tissue neoplasms with genetic data. Am J Surg Pathol. 2005;29:1340–7. 16160477 10.1097/01.pas.0000170343.09562.39

[5] Ricciotti RW Baraff AJ Jour G. High amplification levels of MDM2 and CDK4 correlate with poor outcome in patients with dedifferentiated liposarcoma: a cytogenomic microarray analysis of 47 cases. Cancer Genet. 2017;218-219:69–80. 29153098 10.1016/j.cancergen.2017.09.005

[6] Coindre JM Pédeutour F Aurias A. Well-differentiated and dedifferentiated liposarcomas. Virchows Arch. 2010;456:167–79. 19688222 10.1007/s00428-009-0815-x

[7] Casier J Timmermans I Laenen A. Clinical course and prognostic factors of patients with dedifferentiated liposarcoma: a retrospective analysis. BMC Cancer. 2025;25:517. 40119312 10.1186/s12885-025-13813-w PMC11927263

[8] Nakata E Kunisada T Hasei J. What are the results of resection of localized dedifferentiated liposarcomas in the extremities? Clin Orthop Relat Res. 2020;478:2550–61. 33112583 10.1097/CORR.0000000000001338 PMC7594912

[9] Yamada Y Wakamatsu T Imura Y. Efficacy of surgery in the management of multiple recurrences of retroperitoneal dedifferentiated liposarcoma. World J Surg Oncol. 2024;22:265. 39369260 10.1186/s12957-024-03552-w PMC11452970

[10] Paik B Seo CJ Tan JW. A systematic review of margin status in retroperitoneal liposarcomas: does the R0 margin matter? Front Oncol. 2022;12:891710. 36033535 10.3389/fonc.2022.891710 PMC9404241

[11] Crago AM Dickson MA. Liposarcoma: multimodality management and future targeted therapies. Surg Oncol Clin N Am. 2016;25:761–73. 27591497 10.1016/j.soc.2016.05.007 PMC5010855

[12] Livingston JA Bugano D Barbo A. Role of chemotherapy in dedifferentiated liposarcoma of the retroperitoneum: defining the benefit and challenges of the standard. Sci Rep. 2017;7:11836. 28928422 10.1038/s41598-017-12132-w PMC5605500

[13] Tirumani SH Tirumani H Jagannathan JP. Metastasis in dedifferentiated liposarcoma: predictors and outcome in 148 patients. Eur J Surg Oncol. 2015;41:899–904. 25659772 10.1016/j.ejso.2015.01.012

[14] Brennan MF Antonescu CR Moraco N Singer S. Lessons learned from the study of 10,000 patients with soft tissue sarcoma. Ann Surg. 2014;260:416–21; discussion 421. 25115417 10.1097/SLA.0000000000000869 PMC4170654

[15] Muratori F Frenos F Bettini L. Liposarcoma: clinico-pathological analysis, prognostic factors and survival in a series of 307 patients treated at a single institution. J Orthop Sci. 2018;23:1038–44. 30007495 10.1016/j.jos.2018.06.008

[16] Vos M Koseła-Paterczyk H Rutkowski P. Differences in recurrence and survival of extremity liposarcoma subtypes. Eur J Surg Oncol. 2018;44:1391–7. 29673808 10.1016/j.ejso.2018.03.028

[17] Lahat G Anaya DA Wang X Tuvin D Lev D Pollock RE. Resectable well-differentiated versus dedifferentiated liposarcomas: two different diseases possibly requiring different treatment approaches. Ann Surg Oncol. 2008;15:1585–93. 18398663 10.1245/s10434-007-9805-x

[18] Lee ATJ Thway K Huang PH Jones RL. Clinical and molecular spectrum of liposarcoma. J Clin Oncol. 2018;36:151–9. 29220294 10.1200/JCO.2017.74.9598 PMC5759315

[19] Munoz P Bretcha-Boix P Artigas V Asencio JM. Surgical principles of primary retroperitoneal sarcoma in the era of personalized treatment: a review of the frontline extended surgery. Cancers (Basel). 2022;14:4091. 36077627 10.3390/cancers14174091 PMC9454716

[20] Pascual JMA Hernandez JAF Fernandez GB. Update in pelvic and retroperitoneal sarcoma management: the role of compartment surgery. Cirugía Española (English Edition). 2019;97:480–8. 10.1016/j.ciresp.2019.06.011 31521244

[21] Garcia-Ortega DY. Comprehensive treatment strategy for improving surgical resection rate of retroperitoneal sarcomas: a histology-specific approach narrative review. Front Oncol. 2024;14:2024. 10.3389/fonc.2024.1432900 PMC11491436 39435281

[22] Bonvalot S Gronchi A Le Péchoux C. Preoperative radiotherapy plus surgery versus surgery alone for patients with primary retroperitoneal sarcoma (EORTC-62092: STRASS): a multicentre, open-label, randomised, phase 3 trial. Lancet Oncol. 2020;21:1366–77. 32941794 10.1016/S1470-2045(20)30446-0

[23] Dickson MA Tap WD Keohan ML. Phase II trial of the CDK4 inhibitor PD0332991 in patients with advanced CDK4-amplified well-differentiated or dedifferentiated liposarcoma. J Clin Oncol. 2013;31:2024–8. 23569312 10.1200/JCO.2012.46.5476 PMC3661937

[24] Gounder MM Razak AA Somaiah N. Selinexor in advanced, metastatic dedifferentiated liposarcoma: a multinational, randomized, double-blind, placebo-controlled trial. J Clin Oncol. 2022;40:2479–90. 35394800 10.1200/JCO.21.01829 PMC9467680

[25] Blay JY Honoré C Stoeckle E. Surgery in reference centers improves survival of sarcoma patients: a nationwide study. Ann Oncol. 2019;30:1143–53. 31081028 10.1093/annonc/mdz124 PMC6637376

[26] de Jonge M de Weger VA Dickson MA. A phase I study of SAR405838, a novel human double minute 2 (HDM2) antagonist, in patients with solid tumours. Eur J Cancer. 2017;76:144–51. 28324749 10.1016/j.ejca.2017.02.005

[27] Pollack SM He Q Yearley JH. T-cell infiltration and clonality correlate with programmed cell death protein 1 and programmed death-ligand 1 expression in patients with soft tissue sarcomas. Cancer. 2017;123:3291–304. 28463396 10.1002/cncr.30726 PMC5568958

