# Supplementary material for: Predictors of survival in dedifferentiated liposarcoma: A population-based analysis of the SEER database
Source: Medicine (Baltimore). 2026 Feb 28;105(9):e47738. doi: 10.1097/MD.0000000000047738 (PMC12956179; doi:10.1097/MD.0000000000047738)
Supplement: Supplementary file 1 [file medi-105-e47738-s001.docx]

**Supplementary Table 1. Comparison of Retroperitoneal vs Non-Retroperitoneal Tumors**

| **Characteristic** | **Retroperitoneal**  **(n=1,431)** | **Non-retroperitoneal**  **(n=2,531)** | **P-value** |
| --- | --- | --- | --- |
| Age, median (IQR) | 65 (56-73) | 67 (57-76) | <0.001 |
| Male sex | 894 (62.5%) | 1,780 (70.3%) | <0.001 |
| Tumor size, mean (SD), mm | 205.0 (126.2) | 144.5 (111.1) | <0.0001 |
| Surgery performed | 1,256 (87.8%) | 2,127 (84.0%) | 0.002 |
| Radiation given | 353 (24.7%) | 976 (38.6%) | <0.0001 |
| Chemotherapy given | 282 (19.7%) | 438 (17.3%) | 0.066 |
| Deaths (overall) | 837 (58.5%) | 1,388 (54.8%) | 0.028 |
| Median OS, months | 46.0 | 60.0 | 0.0001 |
| Median CSS, months | 84.0 | 122.0 | <0.0001 |

*OS = overall survival; CSS = cancer-specific survival*
